# Protein Kinase A (PKA) Phosphorylation of Shp2 Protein Inhibits Its Phosphatase Activity and Modulates Ligand Specificity

**DOI:** 10.1074/jbc.M115.642983

**Published:** 2015-03-23

**Authors:** Brian T. Burmeister, Li Wang, Matthew G. Gold, Randal A. Skidgel, John P. O'Bryan, Graeme K. Carnegie

**Affiliations:** From the ‡ Department of Pharmacology,; ¶ University of Illinois Cancer Center, and; ‖ Center for Cardiovascular Research, College of Medicine, University of Illinois at Chicago, Chicago, Illinois, 60612,; § Department of Neuroscience, Physiology, and Pharmacology, University College London, London WC1E 6BT, United Kingdom, and; the ** Jessie Brown Veterans Affairs Medical Center, Chicago, Illinois, 60612

**Keywords:** A-kinase Anchoring Protein (AKAP), Cardiac Hypertrophy, PKA, Signal Transduction, Tyrosine-protein Phosphatase (Tyrosine Phosphatase)

## Abstract

Pathological cardiac hypertrophy (an increase in cardiac mass resulting from stress-induced cardiac myocyte growth) is a major factor underlying heart failure. Src homology 2 domain-containing phosphatase (Shp2) is critical for cardiac function because mutations resulting in loss of Shp2 catalytic activity are associated with congenital cardiac defects and hypertrophy. We identified a novel mechanism of Shp2 inhibition that may promote cardiac hypertrophy. We demonstrate that Shp2 is a component of the protein kinase A anchoring protein (AKAP)-Lbc complex. AKAP-Lbc facilitates PKA phosphorylation of Shp2, which inhibits Shp2 phosphatase activity. We identified two key amino acids in Shp2 that are phosphorylated by PKA. Thr-73 contributes a helix cap to helix αB within the N-terminal SH2 domain of Shp2, whereas Ser-189 occupies an equivalent position within the C-terminal SH2 domain. Utilizing double mutant PKA phosphodeficient (T73A/S189A) and phosphomimetic (T73D/S189D) constructs, *in vitro* binding assays, and phosphatase activity assays, we demonstrate that phosphorylation of these residues disrupts Shp2 interaction with tyrosine-phosphorylated ligands and inhibits its protein-tyrosine phosphatase activity. Overall, our data indicate that AKAP-Lbc integrates PKA and Shp2 signaling in the heart and that AKAP-Lbc-associated Shp2 activity is reduced in hypertrophic hearts in response to chronic β-adrenergic stimulation and PKA activation. Therefore, although induction of cardiac hypertrophy is a multifaceted process, inhibition of Shp2 activity through AKAP-Lbc-anchored PKA is a previously unrecognized mechanism that may promote this compensatory response.

## Teaser

**Background:** The AKAP-Lbc scaffold coordinates cardiac hypertrophic signaling.

**Results:** PKA phosphorylation of Shp2 at Thr-73/Ser-189 inhibits tyrosine-phosphorylated ligand binding and PTP activity.

**Conclusion:** AKAP-Lbc integrates PKA and Shp2 signaling, and AKAP-Lbc-associated Shp2 activity is reduced in hypertrophic hearts following chronic β-adrenergic stimulation.

**Significance:** PKA inhibition of AKAP-Lbc-anchored Shp2 activity is a previously unrecognized mechanism that may promote cardiac hypertrophy.

## Introduction

Localized regulation and integration of intracellular signal transduction is critical for cardiac function. A pivotal mechanism that regulates signal transduction pathways is the formation of complexes between signaling molecules and scaffold proteins (1). Protein kinase A anchoring proteins (AKAPs)^4^ are a diverse family of scaffold proteins that provide a framework for the formation of multienzyme signaling complexes that integrate cAMP signaling with other pathways (2, 3). All members of the AKAP family possess a conserved PKA amphipathic anchoring helix (4, 5) as well as binding sites for additional signaling components (6). Importantly, these scaffold proteins target unique signaling complexes to discrete subcellular locations, thereby generating substrate specificity (2). In the heart, AKAPs play an essential role by integrating PKA signaling with additional enzymes to modulate physiological and pathophysiological processes, including cardiac remodeling and the development of heart failure (7, 8).

Spatiotemporal regulation of signal transduction is integral for proper cardiac function, and perturbation of this regulation can lead to heart failure. AKAPs play a crucial role in directing PKA to several substrates important for cardiac function (6, 9, 10). Previous studies have shown that differential expression of AKAPs may be a critical factor in the development of heart failure (11). One such AKAP, AKAP-Lbc, is encoded by the *AKAP13* gene long transcript and is predominantly expressed in the heart (12). Its expression in the rat heart is induced under hypertrophic conditions, suggesting an important role in cardiac hypertrophy (13). A similar increase in AKAP-Lbc expression has been observed in heart samples from patients with hypertrophic cardiomyopathy compared with control, age-matched, healthy samples (13). Furthermore, our previous work revealed that AKAP-Lbc promotes cardiac hypertrophy through activation of a protein kinase D1 (PKD1)-mediated signaling pathway (13).

Cardiac myocytes respond primarily to an increased workload by increasing in size (hypertrophy). Cardiac hypertrophy is a means to decrease ventricular wall tension and increase cardiac output and stroke volume. Initially, hypertrophy is a beneficial compensatory process. However, prolonged hypertrophy is maladaptive, with the myocardium transitioning to decompensation and cardiac failure. Multiple pathological hypertrophic pathways converge on a set of transcriptional regulators to activate hypertrophic gene expression. Initiation of this developmental gene-reprogramming paradigm is often termed the fetal gene response (14). These “fetal” cardiac genes encode proteins involved in contraction, calcium handling, and metabolism, and their activation accompanies cardiac hypertrophy (15, 16). Therefore, defining the signaling events orchestrated by AKAP-Lbc may lead to the identification of new pharmacological approaches for the treatment of heart failure.

In addition to demonstrating that AKAP-Lbc mobilizes a prohypertrophic signaling pathway through PKD1, we also demonstrated the importance of AKAP-Lbc-tethered PKA in the induction of cardiac hypertrophy through knockdown/rescue experiments (13). Furthermore, we have shown that the protein-tyrosine phosphatase Shp2 (PTPN11) interacts with AKAP-Lbc and demonstrated that AKAP-Lbc integrates PKA and Shp2 signaling in the heart (17). Interestingly, Shp2 is also associated with the modulation of myocyte size, cardiomyopathy, and heart failure (18–20). LEOPARD syndrome patients most commonly manifest congenital heart defects and cardiac hypertrophy because of mutations in the *PTPN11* gene encoding Shp2 that generally result in impaired Shp2 catalytic activity (21, 22). Furthermore, cardiomyocyte-specific genetic deletion of Shp2 results in rapid development of dilated cardiomyopathy (23). Previously, we observed diminished Shp2 activity associated with AKAP-Lbc following chronic isoproterenol treatment, which activates PKA and induces cardiac hypertrophy (17). However, prior to this study, the mechanism by which AKAP-Lbc inhibits Shp2 was unknown. Here we report that AKAP-Lbc facilitates PKA phosphorylation of Shp2 at amino acid residues Thr-73 and Ser-189, thereby inhibiting its protein tyrosine phosphatase (PTP) activity and disrupting its binding to tyrosine-phosphorylated ligands. Although induction of cardiac hypertrophy is a multifaceted process, inhibition of Shp2 activity through enhanced PKA signaling represents a previously unrecognized mechanism contributing to cardiac hypertrophy.

## EXPERIMENTAL PROCEDURES

### 

#### 

##### Antibodies and Reagents

Anti-GFP antibody (mouse, 1:1000) was from Clontech. Anti-FLAG M2 antibody (mouse, 1:1000) and anti-α-actinin (mouse, 1:500) were from Sigma. Anti-phospho-PKA substrate (RR*X*S*/T*) antibody (rabbit, 1:1000) was from Cell Signaling Technology. Anti-phosphotyrosine antibody, clone 4G10 (mouse, 1:1000), and anti-GST (mouse, 1:1000) were from EMD Millipore. Wild-type Shp2 mammalian expression constructs were provided by Dr. Gen-Sheng Feng (University of California, San Diego). Mammalian expression constructs for GAB1, GAB2, and constitutively active Src (Y527F) were provided by Dr. Andrei Karginov (University of Illinois at Chicago).

##### Bacterial Expression

GST and GST-Shp2-SH2 domain proteins were expressed as N-terminal GST-tagged fusions using the pGEX-4T1 vector (Amersham Biosciences) in bacteria (DH5α) and purified by glutathione-Sepharose chromatography (GE Healthcare). Bacterial cultures were grown overnight at 37 °C. The following day, bacterial cultures were diluted 1:10, grown to an *A*_600_ of 0.6, and induced with 0.1 mm isopropyl 1-thio-β-d-galactopyranoside for 3 h at 37 °C. Cells were resuspended in MTPBS (16 mm Na_2_HPO_4_, 4 mm NaH_2_PO_4_-H_2_O, 150 mm NaCl, 50 mm EDTA, and 1% TritonX-100 (pH 7.3)) and sonicated, and then insoluble debris was removed by centrifugation. The supernatant was then incubated with glutathione-Sepharose beads (GE Healthcare) for 1 h at 4 °C. Beads were then washed three times with MTPBS.

##### Transfections, Coimmunoprecipitations, and Pulldowns

HEK293T cells were transfected and lysed in cell lysis buffer (10 mm sodium phosphate buffer (pH 6.95), 150 mm NaCl, 5 mm EDTA, 5 mm EGTA, and 1% Triton X-100) as described previously (24). For phosphorylation experiments, the phosphatase inhibitor microcystin-LR was included (100 nm). Lysates were incubated on ice for 10 min and centrifuged at 20,000 × *g* for 15 min at 4 °C. Cleared lysates were incubated with antibodies for 1 h at 4 °C with rocking, followed by precipitation of antibody-antigen complexes with protein A/G-agarose. Immunoprecipitates were washed 5 × 1 ml in lysis buffer, eluted in SDS-PAGE sample buffer, and separated by SDS-PAGE. GST pulldowns were performed similarly, except that protein complexes were isolated by incubation with glutathione-Sepharose for 1 h at 4 °C.

##### Cell Culture, Immunocytochemistry, and Cell Imaging

Preparation of primary neonatal rat ventricular myocytes and confocal microscopy experiments were performed as described previously (13). Neonatal rat ventricular myocytes were electroporated using a modified Amaxa Nucleofector protocol. After incubation at 37 °C for 48 h post-electroporation, cells were washed twice with PBS and fixed in 3.7% paraformaldehyde in PBS, followed by staining for α-actinin. The secondary antibodies used were from Invitrogen. Confocal images were acquired using a Carl Zeiss LSM 510 mounted on an Axiovert 100 m microscope. Images were obtained using a 488-nm argon laser for GFP and 561 nm for red fluorescent protein with a Plan-Apochromat ×63/1.4 oil immersion objective lens. The cell area of α-actinin and GFP double-positive myocytes was quantified using ImageJ as described previously (13).

##### In Vitro PTP Activity Assay

Following immunoprecipitation of either AKAP-Lbc or Shp2, immune complexes were washed five times with IP buffer (10 mm sodium phosphate buffer (pH 6.95), 150 mm NaCl, 5 mm EDTA, 5 mm EGTA, and 1% Triton X-100) before being resuspended in phosphatase assay buffer (50 mm HEPES, 100 mm NaCl, 5 mm DTT, 2 mm Na_2_ EDTA, and 0.01% Brij-35 (pH 7.5)). The phosphatase assay was carried out in a total reaction volume of 50 μl using 30 μm fluorescein diphosphate as substrate. After a 20-min incubation at 30 °C, the supernatant was transferred to a 96-well plate, and phosphatase activity was measured using a PHERAstar FS microplate reader with excitation at 485 nm and emission at 520 nm. For calibration of PTP activity using this assay, T cell PTP (New England Biolabs) was serially diluted and used for assay as described above. Fluorescence intensity was measured for known amounts of enzyme ranging from 0–500 milliunits of specific activity. One unit is defined as the amount of enzyme that hydrolyzes 1 nmol of *p-*nitrophenyl phosphate (50 mm) in 1 min at 30 °C in a total reaction volume of 50 μl.

##### Structural Analysis

Structural alignments were performed using the secondary structure matching superimpose tool in Coot (25). Three-dimensional schematic representations of SHP-2 were generated using PyMOL (Schroedinger LLC).

##### Statistical Analysis of Data

All data are expressed as mean ± S.E. Differences in quantitative variables were examined by one-way analysis of variance or an unpaired two-tailed Student's *t* test. *p* < 0.05 was considered significant (*, *p* < 0.05; **, *p* < 0.01; ***, *p* < 0.001). All analyses were performed using InStat.

## RESULTS

### 

#### 

##### AKAP-Lbc-associated Shp2 Activity Is Increased under Basal Conditions

**FIGURE 1.**
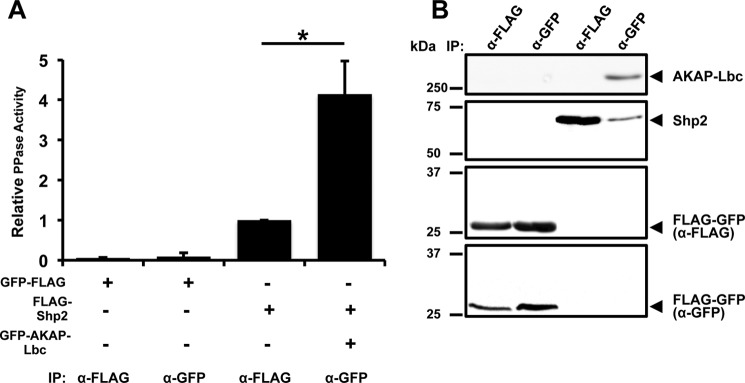
**The specific activity of AKAP-Lbc-associated Shp2 is increased under basal conditions.** *A*, HEK293T cells were transfected with FLAG-Shp2, GFP-AKAP-Lbc plus FLAG-Shp2, or FLAG-GFP vector as a control. Shp2 or AKAP-Lbc was isolated from cell lysates using anti-FLAG-antibody or anti-GFP-antibody, respectively. Immunoprecipitates were washed, and PTP activity was measured by a fluorometric *in vitro* assay. Parallel control anti-FLAG and anti-GFP IPs were assayed using an equal amount of lysate (2 mg) from cells expressing FLAG-GFP vector. Results show mean PTP activity per IP ± S.E. after normalizing to the amount of Shp2 in the IP. Band intensities were quantified using ImageJ and were within the linear dynamic range of detection. All assays were performed in triplicate for three independent experiments. Differences in quantitative variables were examined by analysis of variance. *, *p* < 0.05. *B*, Western blots showing corresponding levels of Shp2, AKAP-Lbc, and control FLAG-GFP vector in samples used for PTP activity measurement in *A*.

We have demonstrated previously that Shp2 is a component of the AKAP-Lbc complex (17). Under conditions of enhanced PKA signaling, such as chronic β-adrenergic stimulation or forskolin/IBMX treatment, AKAP-Lbc facilitates PKA phosphorylation of Shp2, which inhibits its PTP activity (17). To determine whether association with AKAP-Lbc affects the specific activity of Shp2 under basal conditions, we performed *in vitro* PTP activity assays and compared the specific activity of total Shp2 *versus* Shp2 associated with AKAP-Lbc. FLAG-tagged Shp2 was coexpressed in HEK293T cells with either FLAG-GFP or GFP-AKAP-Lbc (Fig. 1). Following immunoprecipitation, PTP activity was measured (Fig. 1*A*) and normalized to the amount of Shp2 in the IP (Fig. 1*B*). Similarly, GFP-tagged AKAP-Lbc was immunoprecipitated, and PTP activity was assayed and normalized to the amount of Shp2 present in the IP. The specific PTP activity of AKAP-Lbc-associated Shp2 was increased 4-fold (*p* = 0.045) compared with the total Shp2 immunoprecipitated from whole cell lysate.

##### PKA Phosphorylation of Sites Thr-73 and Ser-189 Inhibits Shp2 Activity

**FIGURE 2.**
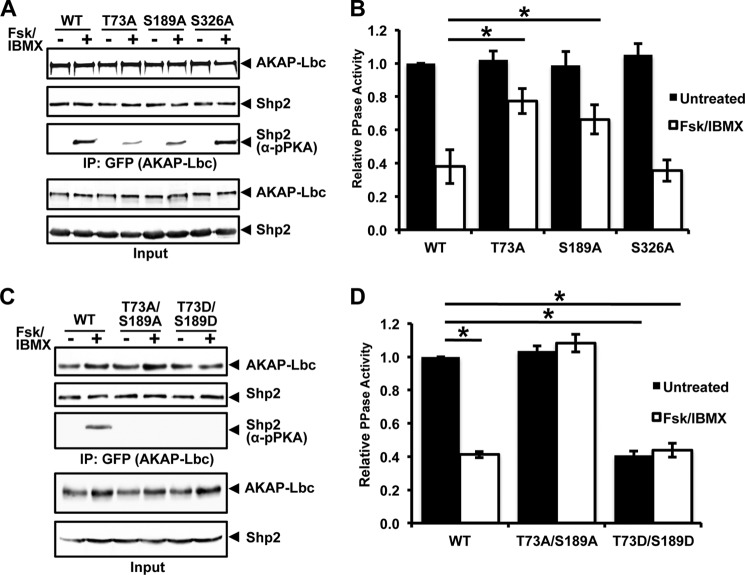
**Phosphorylation of Shp2 residues Thr-73 and Ser-189 by PKA regulates Shp2 activity.** *A*, Scansite was used to identify consensus PKA phosphorylation (R-R-*X-*Ser(P)/Thr(P)) sites in Shp2, yielding three predicted residues: Thr-73, Ser-189, and Ser-326. HEK293T cells were transfected with wild-type or Ser/Thr-to-Ala mutant Shp2 expression constructs. Prior to lysis, cells were treated for 20 min with either dimethyl sulfoxide (*DMSO*, vehicle) or with forskolin (*Fsk*, 20 μm) and IBMX (75 μm) to activate PKA. AKAP-Lbc was isolated from cell lysates using anti-GFP-antibody, and immune complexes were used for *in vitro* measurement of PTP activity. Western blot loading controls demonstrate comparable expression of GFP-AKAP-Lbc and associated FLAG-Shp2-WT and FLAG-Shp2 mutants for all assays. Blots were probed with anti-PKA-phospho-substrate antibody to examine the extent of Shp2 phosphorylation. *B*, quantification of three independent *in vitro* PTP assays. *, *p* < 0.05. *C*, HEK293T cells were transfected with AKAP-Lbc and FLAG-Shp2-WT or FLAG-Shp2 T73A/S189A (PKA phosphodeficient) or FLAG-Shp2 T73D/S189D (PKA phosphomimetic) expression vectors. Prior to lysis, cells were treated for 20 min with either dimethyl sulfoxide (vehicle) or with forskolin/IBMX to activate PKA. AKAP-Lbc was isolated from cell lysates using anti-GFP-antibody, and immune complexes were used for *in vitro* measurement of PTP activity. Western blot loading controls demonstrated comparable expression of AKAP-Lbc and associated Shp2-WT and Shp2 mutants for all assays. Blots were probed with anti-PKA phosphosubstrate antibody to examine the extent of Shp2 phosphorylation. *D*, quantification of three independent *in vitro* PTP assays. *, *p* < 0.05.

To determine which amino acid residues within Shp2 are phosphorylated by PKA, we first identified potential candidate sites on the basis of the consensus PKA phosphorylation motif (R-R-*X*-Ser(P)/Thr(P)) using Scansite 2.0 (26). We identified three candidate PKA phosphorylation sites (Thr-73, Ser-189, and Ser-326) that were individually mutated to alanine. WT or mutant Shp2 was coexpressed with AKAP-Lbc in HEK293T cells, and then cells were treated with either vehicle or forskolin/IBMX for 20 min. Following immunoprecipitation of AKAP-Lbc, phosphorylation of Shp2 was examined using an antibody that recognizes the consensus PKA phosphorylation sites R-R-*X*-Ser(P)/Thr(P). Treatment with forskolin/IBMX resulted in PKA-mediated phosphorylation of Shp2. Importantly, phosphorylation was diminished in the T73A and S189A mutants but not in the S326A mutant (Fig. 2*A*).

To determine the effect of PKA phosphorylation on Shp2 activity, we measured the PTP activity of WT, T73A, S189A, and S326A versions of Shp2 in AKAP-Lbc immunoprecipitates (Fig. 2*B*). Under basal conditions, no change in AKAP-Lbc-associated Shp2 activity was observed after mutation of the candidate sites. Treatment with forskolin/IBMX inhibited AKAP-Lbc-associated WT Shp2 activity as well as that of the S326A mutant. However, mutation of either Thr-73 or Ser-189 to alanine reduced this inhibition 2-fold (*p* = 0.035) and 1.7-fold (*p* = 0.044), whereas mutation of Ser-326 to alanine did not alter the inhibition of Shp2 following PKA activation. This difference in Shp2 activity is not due to changes in the association of the Shp2 mutants with AKAP-Lbc because equivalent amounts of each Shp2 protein were present in the AKAP-Lbc IPs (Fig. 2*A*).

The combined mutation of T73A and S189A in Shp2 eliminated PKA-induced phosphorylation of Shp2 (Fig. 2*C*). Consistent with the lack of PKA phosphorylation, the phosphatase activity of this phosphodefective Shp2 mutant was unaffected following PKA activation (Fig. 2*D*). We also generated a phosphomimetic version of Shp2 by mutation of Thr-73 and Ser-189 to aspartate. Although PKA-induced phosphorylation of the Shp2 T73D/S189D mutant was also impaired, the AKAP-Lbc-associated PTP activity of this phosphomimetic mutant was similar to that of WT Shp2 following PKA activation, regardless of whether PKA was activated in cells (Fig. 2*D*). Therefore, the T73A/S189A mutations block PKA-mediated phosphorylation and inhibition of AKAP-Lbc-associated Shp2, whereas the T73D/S189D phosphomimetic mutations lead to impaired AKAP-Lbc-associated Shp2 activity under basal conditions.

##### Shp2 T73D/S189D SH2 Domains Display Altered Ligand Specificity

**FIGURE 3.**
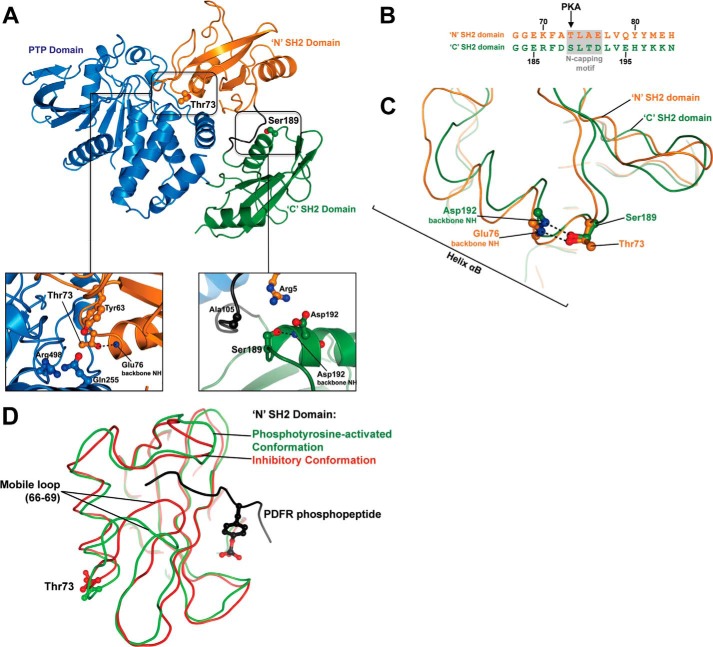
**Modeling PKA phosphorylation of Shp2.** *A*, schematic representation of the full-length Shp2 structure (PDB code 2SHP) showing the location of two PKA phosphorylation sites in relation to the first N SH2 domain (*orange*), the second C SH2 domain (*green*), and the PTP domain (*blue*). The *left box* shows the location of Thr-73. The hydroxyl group of the Thr-73 side chain participates in a helix-capping interaction with the main chain amide group of Glu-76 at the N terminus of helix αB in the N SH2 domain. Three residues are in the vicinity that have the potential to interact with Thr(P)-73: Arg-498 and Gln-255 in the PTP domain and Tyr-63 in the N SH2 domain. The *right box* shows the location of Ser-189. The hydroxyl group of the Ser-189 side chain forms a helix cap with the main-chain amide of Asp-192 at the N terminus of helix αB in the C SH2 domain. Phosphorylation at Ser-189 likely destabilizes two interactions in addition to disrupting the helix cap: a hydrophobic packing interaction between Ser-189 and Ala-105 and a salt bridge between Arg-5 and Asp-192. *B*, sequence alignment of the N and C SH2 domains indicates that Thr-73 and Ser-189 are at equivalent positions within their respective SH2 domains. Both amino acids form the first position of an N-capping motif that precedes helix αB. *C*, superposition of the N (*orange*) and C (*green*) SH2 domains further demonstrates the structural equivalence of the two amino acids. Phosphorylation in both cases can be expected to break the N-capping interaction and lead to destabilization of helix αB. *D*, superposition of the SHP-2 N SH2 domain in the presence (*green*, PDB code 1AYA) and absence (red, PDB code 2SHP) of phosphotyrosine ligand (*black*) indicates that Ser/Thr at the N-terminal cap of helix αB is not highly dynamic. Furthermore, phosphorylation of Thr-73/Ser-189 will not sterically hinder the recruitment of phospholigand.

The location of the PKA phosphorylation sites in relation to the Shp2 N-terminal (N) and C-terminal (C) SH2 domains and the PTP domain are shown in context of the structure of Shp2 (Fig. 3*A*). The hydroxyl group of the Thr-73 side chain participates in a helix-capping interaction with the main-chain amide group of Glu-76 at the N terminus of helix αB in the “N” SH2 domain. Similarly, the hydroxyl group of the Ser-189 side chain forms a helix cap with the main-chain amide of Asp-192 at the N terminus of helix αB in the “C” SH2 domain. Sequence alignment of the N and C SH2 domains demonstrates that Thr-73 and Ser-189 are located at analogous positions within their respective SH2 domains (Fig. 3*B*). The two amino acids are structurally equivalent in that both form the first position of an N-capping motif that precedes helix αB of the SH2 domain (Fig. 3*C*). The most elegant way to explain the effects of dual Thr-73/Ser-189 phosphorylation on Shp2 activity is that phosphorylation in both cases would be expected to break the N-capping interaction and lead to destabilization/melting of helix αB. A stable αB helix is required for phosphotyrosine recruitment (Fig. 3*D*). Therefore, PKA phosphorylation may reduce Shp2 activation by reducing the binding affinity of phosphotyrosine ligands to each SH2 domain by the same mechanism.

**FIGURE 4.**
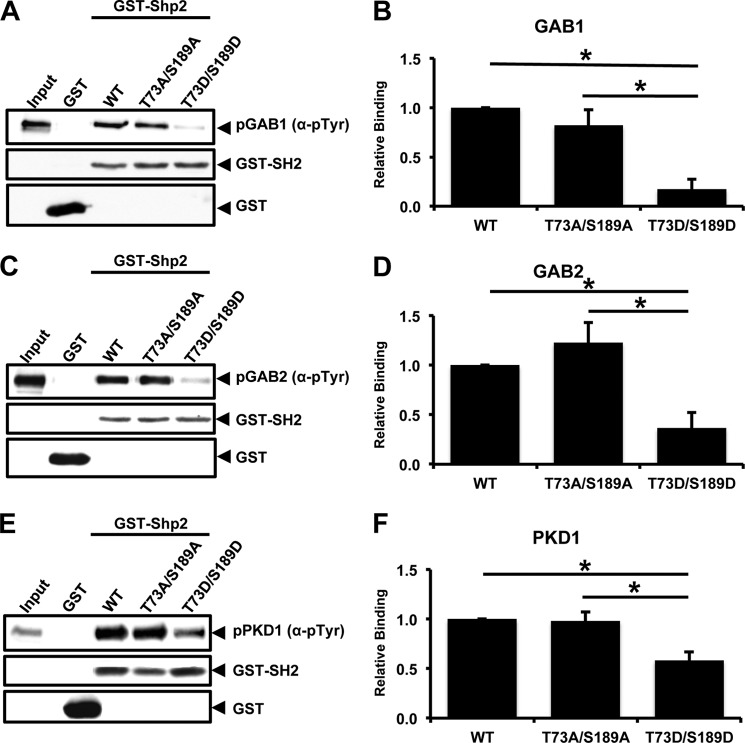
**Mutation of residues Thr-73 and Ser-189 to Asp disrupts Shp2 binding to tyrosine-phosphorylated ligands.** *A*, association between the GAB1 and wild-type Shp2 (*WT*) or PKA phosphodeficient Shp2 (T73A/S189A) or PKA phosphomimetic Shp2 (T73D/S189D) SH2 domains was assessed by pulldown assays using glutathione-Sepharose bead-bound bacterial fusion proteins. GST-Shp2 SH2 proteins were incubated with lysates from HEK293T cells transfected with Venus-GAB1 and constitutively active Src expression vectors. Proteins bound to beads were immunoblotted for phosphotyrosine (α*-*4G10) and GST. *B*, band intensities were quantified using ImageJ, and relative binding was determined from the ratios of phosphorylated Venus-GAB1 to GST-Shp2 SH2 input. *, *p* < 0.05. *C*, the association between GAB2 and wild-type Shp2 or mutant Shp2 SH2 domains was assessed as above. GST-Shp2 SH2 proteins were incubated with lysates from HEK293T cells transfected with Venus-GAB2 and constitutively active Src expression vectors. Proteins bound to beads were immunoblotted for phosphotyrosine and GST. *D*, band intensities were quantified using ImageJ, and relative binding was determined from the ratios of phosphorylated Venus-GAB2 to GST-Shp2 SH2 input. *, *p* < 0.05. *E*, the association between PKD1 and wild-type Shp2 or mutant Shp2 SH2 domains was assessed. GST-Shp2 SH2 proteins were incubated with lysates from HEK293T cells transfected with GFP-PKD1 and constitutively active Src expression vectors. Proteins bound to beads were immunoblotted for phosphotyrosine and GST. *F*, band intensities were quantified using ImageJ, and relative binding was determined from the ratios of phosphorylated GFP-PKD1 to GST-Shp2 SH2 input. *, *p* < 0.05.

To determine whether PKA phosphorylation of Shp2 disrupts binding to tyrosine-phosphorylated ligands, we performed *in vitro* pulldown assays using bacterially expressed WT or mutant (T73A/S189A or T73D/S189D) GST-tagged Shp2 SH2 domains. The scaffolding adaptor proteins Grb2-associtated binding protein 1 (GAB1) and Grb2-associated binding protein 2 (GAB2) as well as PKD1 were coexpressed with constitutively active Src tyrosine kinase in HEK293T cells. Lysates from these cells were subsequently used as inputs for pulldown with GST-SH2. The pulldown experiments demonstrated that the T73A/S189A double mutation has a negligible effect on Shp2 SH2 binding to GAB1 (Fig. 4, *A* and *B*), GAB2 (Fig. 4, *C* and *D*), or PKD1 (Fig. 4, *E* and *F*). Conversely, the T73D/S189D double mutation reduced Shp2 binding to GAB1 5.7-fold (Fig. 4, *A* and *B*; *p* = 0.011), to GAB2 2.7-fold (Fig. 4, *C* and *D*; *p* = 0.025), and to PKD1 1.7-fold (Fig. 4, *E* and *F*; *p* = 0.028).

##### The Shp2-T73A/S189A Mutant Inhibits Isoproterenol-induced Tyrosine Phosphorylation of PKD1 in the AKAP-Lbc Complex

**FIGURE 5.**
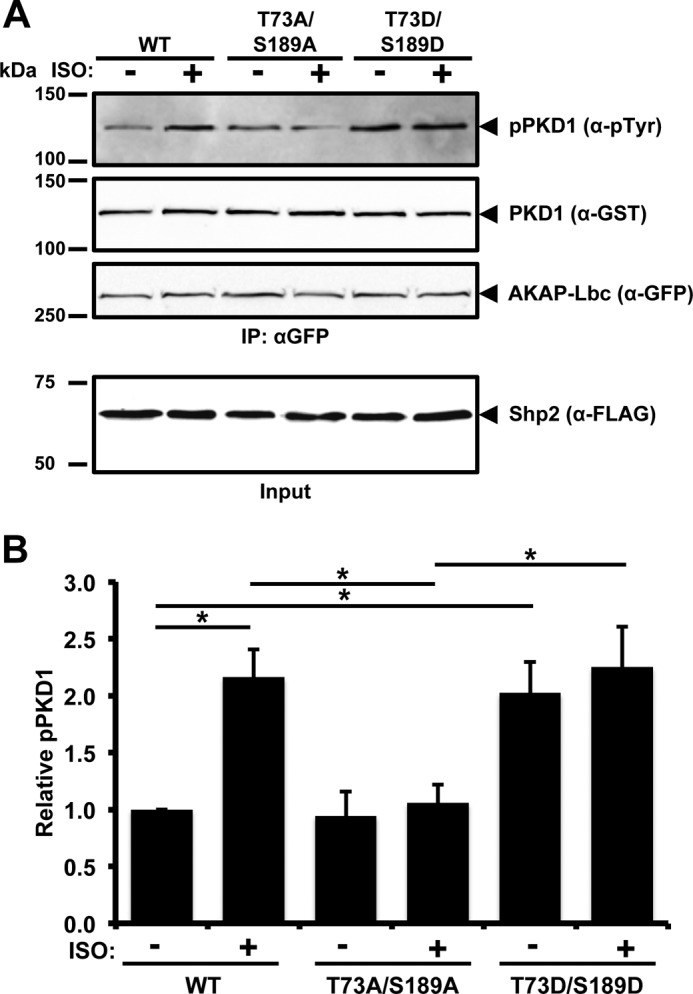
**Inhibition of Shp2 by PKA enhances tyrosine phosphorylation of PKD1 in the AKAP-Lbc complex.** *A*, HEK293 cells stably expressing the β1-adrenergic receptor were transfected with the GFP-AKAP-Lbc, GST-PKD1, and either FLAG-Shp2-WT or FLAG-Shp2 T73A/S189A or FLAG-Shp2 T73D/S189D expression vectors. Prior to lysis, cells were treated for 20 min with either dimethyl sulfoxide (vehicle) or ISO (10 μm) to activate PKA. AKAP-Lbc was isolated from cell lysates using anti-GFP-antibody. IPs were washed, and the bound proteins were separated by SDS-PAGE and transferred to nitrocellulose. Detection of tyrosine-phosphorylated GST-PKD1, total GST-PKD1, GFP-AKAP-Lbc, and FLAG-Shp2 was carried out by immunoblotting. *B*, band intensities were quantified from four independent experiments using ImageJ, and relative tyrosine-phosphorylation of GST-PKD1 was determined from the ratio of tyrosine-phosphorylated GST-PKD1 to total GST-PKD1 in the IP. *, *p* < 0.05.

It has been reported that the tyrosine kinases Src and Abl phosphorylate PKD1 (27–29), suggesting that tyrosine phosphorylation within the pleckstrin homology domain of PKD1 induces a conformational change, promoting PKCδ recruitment and leading to activation of PKD1. To determine whether isoproterenol (ISO)-stimulated PKA activation results in enhanced tyrosine phosphorylation of PKD1 by inhibiting Shp2 in the AKAP-Lbc complex, we utilized HEK293 cells stably expressing the β1-adrenergic receptor (30). These cells were transiently transfected with vectors encoding AKAP-Lbc, PKD1, and either Shp2 WT, Shp2-T73A/S189A, or Shp2-T73D/S189D. Analysis of AKAP-Lbc-associated PKD1 tyrosine phosphorylation revealed that Shp2 WT enhanced ISO-induced PKD1 phosphorylation. Interestingly, expression of the Shp2-T73A/S189A mutant abolished the effect of ISO stimulation on PKD1 phosphorylation (Fig. 5). In contrast, expression of the Shp2-T73D/S189D mutant enhanced basal tyrosine phosphorylation of PKD1 in the AKAP-Lbc complex 2-fold (*p* = 0.014), and treatment with ISO did not further increase the level of PKD1 phosphorylation (Fig. 5).

##### Overexpression of Shp2 T73A/S189A Inhibits Isoproterenol-induced Cardiac Hypertrophy

**FIGURE 6.**
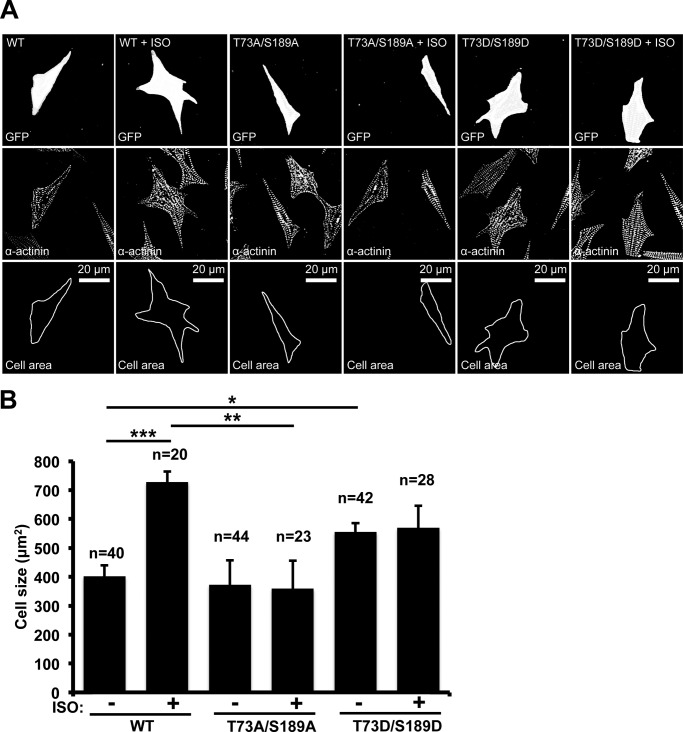
*A*, neonatal rat ventricular myocytes were transfected with a GFP-tagged wild-type Shp2 (*WT*), PKA phosphodeficient Shp2 (T73A/S189A), or PKA phosphomimetic Shp2 (T73D/S189D) expression vector and then treated with either dimethyl sulfoxide or ISO for 48 h to induce hypertrophy. Cells were fixed, permeabilized, and stained with α-actinin for cell imaging. Representative images are shown. *B*, cardiomyocytes with both α-actinin staining and GFP expression were selected for cell size measurements and quantified. Data are expressed as mean ± S.E. The number of cells counted is indicated.

Chronic β-adrenergic receptor stimulation by ISO was used as an *in vitro* cellular model to study hypertrophic PKA signaling. Neonatal rat ventricular myocytes were transfected with GFP-tagged vectors expressing either Shp2-WT, Shp2-T73A/S189A, or Shp2-T73D/S189D. In myocytes expressing GFP-Shp2-WT, treatment with ISO increased cellular size 1.8-fold (Fig. 6, *p* < 0.001). This hypertrophic effect of ISO was inhibited by expression of Shp2-T73A/S189A. However, expression of the Shp2 phosphomimetic mutant Shp2-T73D/S189D increased myocyte size under basal conditions 1.38-fold (*p* = 0.011) with no further increase in size after ISO treatment (Fig. 6).

## DISCUSSION

We have demonstrated previously that Shp2 is a component of the AKAP-Lbc complex and that AKAP-Lbc facilitates PKA phosphorylation of Shp2 (17). In this report, we identify two key amino acids in Shp2 that are phosphorylated by PKA, Thr-73 and Ser-189. We demonstrate that phosphorylation of these residues disrupts Shp2 interaction with tyrosine-phosphorylated ligands and inhibits its protein-tyrosine phosphatase activity. Furthermore, our data indicate that the phosphomimetic mutant of Shp2, Shp2-T73D/S189D, induces a significant increase in the size of neonatal rat cardiomyocytes in the absence of ISO stimulation.

Our previous mapping experiments have shown that Shp2 binds predominately to a central region of AKAP-Lbc (17). Under conditions of enhanced PKA activity, Shp2 association with the AKAP-Lbc complex facilitates PKA phosphorylation and inhibition of Shp2 activity (17). This study demonstrates that, under basal conditions, Shp2-specific activity is enhanced within the AKAP-Lbc complex, indicating that Shp2 binding to AKAP-Lbc increases its activity. Taken together, these findings suggest that the AKAP-Lbc complex plays a critical role in regulating Shp2 activity and may mediate a negative feedback mechanism under conditions of increased PKA activity.

Studies of AKAP-Lbc-associated activity of WT and Shp2 mutants revealed the criticality of residues 73 and 189. Mutation of Thr-73/Ser-189 to Ala or Asp abolished phosphorylation by PKA. Additionally, the T73A/S189A mutations block PKA-mediated inhibition of Shp2 activity, whereas the phosphomimetic mutations (T73D/S189D) lead to impaired AKAP-Lbc-associated Shp2 activity under basal conditions. Together, these results indicate that sites Thr-73 and Ser-189 are phosphorylated by PKA, inhibiting Shp2 activity. Further investigation of the mechanism of PKA-mediated Shp2 inhibition demonstrated that the T73A/S189A mutations do not significantly alter Shp2 SH2 binding to phosphorylated GAB1, GAB2, and PKD1. However, the phosphomimetic T73D/S189D mutations resulted in impaired phosphotyrosine binding by the Shp2 SH2 domains, suggesting that PKA phosphorylation of Shp2 modifies its ligand specificity.

We propose that dual Thr-73/Ser-189 phosphorylation affects Shp2 activity by a mechanism in which phosphorylation at both sites disrupts N-capping interactions with the SH2 domains, leading to destabilization/melting of helix αB. A stable αB helix is required for phosphotyrosine recruitment by the SH2 domains. Therefore, PKA phosphorylation may inhibit Shp2 activation by reducing the binding affinity of phosphotyrosine ligands to each SH2 domain by a similar mechanism. Interestingly, Thr-73 is inaccessible when Shp2 maintains an inactive conformation (31). Therefore, phosphorylation by PKA at this site would presumably only be possible following activation of the phosphatase, which, as we demonstrated here, occurs within the AKAP-Lbc complex under basal conditions.

Another potential contributory mechanism is that Thr(P)-73 interacts with a phosphobinding site on the PTP domain, including residues Arg-498 and Gln-255, thereby stabilizing Shp2 in its inactive conformation. Additionally, phosphorylation of Ser-189 may disrupt interactions that link the N and C SH2 domains to reduce cooperative binding of phosphotyrosine ligands to the two SH2 domains.

Interestingly, mutation of residue Thr-73 to Ile has been observed in Noonan syndrome and juvenile myelomonocytic leukemia patients (32). The T73I mutation leads to higher basal Shp2 activity by shifting the basal equilibrium constant toward the active state. Activation by insulin receptor substrate 1 (IRS-1) is not greatly affected (33). This suggests that the branched aliphatic side chain of Ile cannot be accommodated in the pocket of the phosphatase domain in proximity to Tyr-63, Arg-498 and Gln-255. Both branches of the Ile side chain extend to the δ position, whereas both Thr and Thr(P) have one branch terminating earlier with a methyl group at the γ position. Ile-73, therefore, likely disrupts packing between the N-terminal SH2 domain and the phosphatase domain.

PKA, AKAP-Lbc, and Shp2 play prominent roles in signaling mechanisms leading to the induction of cardiac hypertrophy. Mutations resulting in loss of Shp2 catalytic activity are associated with congenital heart defects and cardiac hypertrophy (21, 22). AKAP-Lbc is also implicated in cardiac hypertrophic signaling, and we have shown that AKAP-Lbc-tethered PKA is important for the induction of cardiomyocyte hypertrophy (13). Consistent with the observation that mice deficient in PKA-Cβ are resistant to hypertrophic stimuli (34), current data from our laboratory indicate that specific PKA inhibition abolishes ISO-induced cardiomyocyte hypertrophy, demonstrating the primary contribution of PKA (35). The results of this study demonstrate that the hypertrophic effect of ISO is inhibited by expression of the Shp2 T73A/S189A mutant, whereas expression of the T73D/S189D mutant significantly increases myocyte size in the absence of ISO stimulation. These results suggest that PKA phosphorylation of Shp2 at Thr-73 and Ser-189 contributes to β-adrenergic receptor-induced cardiomyocyte hypertrophy.

We have demonstrated previously that AKAP-Lbc facilitates the activation of PKD1 in response to hypertrophic stimuli (24). Activated PKD1 then phosphorylates class II histone deacetylases to promote their nuclear export, leading to the derepression of the transcription factor MEF2, resulting in cardiac hypertrophy and tissue remodeling through MEF2-dependent transcription of hypertrophic genes (13). Others have shown that PKD1 conditional knockout mice are resistant to ISO-dependent hypertrophy (36). Moreover, it has been reported that tyrosine phosphorylation within the pleckstrin homology domain of PKD1 enhances its catalytic activity (29). Here we demonstrate that ISO stimulates tyrosine phosphorylation of AKAP-Lbc-associated PKD1 in HEK293 cells stably expressing the β1-adrenergic receptor. The Shp2 T73A/S189A mutant inhibits ISO-induced PKD tyrosine phosphorylation, whereas the Shp2 phosphomimetic mutant increases the basal tyrosine phosphorylation of PKD1 in the AKAP-Lbc complex. Taken together, our data suggest that Shp2 in the AKAP-Lbc complex maintains PKD in the dephosphorylated state and that, upon PKA activation, AKAP-Lbc facilitates PKA phosphorylation and inhibition of Shp2, thereby allowing enhanced PKD1 tyrosine phosphorylation and activation.

In conclusion, we identified a novel mechanism of Shp2 inhibition that may promote cardiac hypertrophy. AKAP-Lbc facilitates PKA phosphorylation of Shp2, which inhibits Shp2 phosphatase activity. We identified two critical amino acids in Shp2 that are phosphorylated by PKA. We demonstrate that dual phosphorylation of Thr-73/Ser-189 disrupts Shp2 interaction with tyrosine-phosphorylated ligands and inhibits its protein tyrosine phosphatase activity. Interestingly, we and others have observed up-regulation of AKAP-Lbc expression under hypertrophic conditions (13, 17, 37). We speculate that this increase in AKAP-Lbc levels may facilitate the integration of PKA, Shp2, and PKD1 signaling in the heart. Although induction of cardiac hypertrophy is a multifaceted process, inhibition of Shp2 activity through AKAP-Lbc-anchored PKA is a previously unrecognized mechanism that may promote cardiac hypertrophy in response to chronic β-adrenergic stimulation.

## Footnotes

4 The abbreviations used are:

AKAP: A-kinase-anchoring protein
PTP: protein-tyrosine phosphatase
IP: immunoprecipitate(s)
IBMX: isobutylmethylxanthine
ISO: isoproterenol.
